# Circulating dengue virus serotypes and vertical transmission in *Aedes* larvae during outbreak and inter-outbreak seasons in a high dengue risk area of Sri Lanka

**DOI:** 10.1186/s13071-021-05114-5

**Published:** 2021-12-23

**Authors:** Chandana Wijesinghe, Jagath Gunatilake, P. H. D. Kusumawathie, P. D. N. N. Sirisena, S. W. P. L. Daulagala, Bushran N. Iqbal, Faseeha Noordeen

**Affiliations:** 1 Teaching Hospital Peradeniya, Peradeniya, 20400 Sri Lanka; 2 Postgraduate Institute of Science, University of Peradeniya, Peradeniya, 20400 Sri Lanka; 3 Department of Geology, Faculty of Science, University of Peradeniya, Peradeniya, 20400 Sri Lanka; 4 Regional Office, Anti-Malaria Campaign, Kandy, 20000 Sri Lanka; 5 Department of Microbiology, Faculty of Medicine, University of Peradeniya, Peradeniya, 20400 Sri Lanka

**Keywords:** Dengue, Dengue viruses, Vertical transmission, *Aedes albopictus*, *Aedes aegypti*

## Abstract

**Background:**

Spatial and temporal changes in the dengue incidence are associated with multiple factors, such as climate, immunity among a population against dengue viruses (DENV), circulating DENV serotypes and vertical transmission (VT) of DENV in an area at a given time. The level of VT in a specific location has epidemiological implications in terms of viral maintenance in vectors. Identification of the circulating DENV serotypes in both patients and *Aedes* mosquito larvae in an area may be useful for the early detection of outbreaks. We report here the results of a prospective descriptive study that was conducted to detect the levels of VT in *Aedes* mosquito larvae and circulating DENV serotypes in patients and *Aedes* mosquito larvae from December 2015 to March 2017 in an area of Sri Lanka at high risk for dengue.

**Methods:**

A total of 200 patients with clinically suspected dengue who had been admitted to a tertiary care hospital during a dengue outbreak (3 study periods: December 2015–January 2016, June–August 2016, December 2016–January 2017) and in the inter-outbreak periods (February–May 2016 and September–November 2016) were investigated. Blood samples were drawn from the study participants to test for DENV. The houses of the study participants were visited within 7 days of admission to the hospital, and *Aedes* larvae were also collected within a radius of 400 m from the houses. The larvae were separately identified to species and then pooled according to each patient’s identification number. Patients’ sera and the *Aedes* larvae were tested to identify the infecting DENV serotypes using a reverse transcription PCR (RT-PCR) method. Levels of VT in *Aedes* mosquito larvae were also identified.

**Results:**

All four DENV serotypes (DENV-1 to -4) were identified in the study area. In the early part of the study (December 2015–February 2016), DENV-3 was predominant and from April 2016 to March 2017, DENV-2 became the most predominant type. Four cases of DENV co-infections were noted during the study period in patients. Interestingly, all four DENV serotypes were detected in *Aedes albopictus* larvae, which was the prominent immature vectorial form identified throughout the study period in the area, showing 9.8% VT of DENV. With the exception of DENV-4, the other three DENV serotypes were identified in *Aedes aegypti* larvae with a VT of 8.1%.

**Conclusion:**

Comparatively high rates of VT of DENV was detected in *Ae. albopictus* and *Ae. aegypti* larvae. A shift in the predominant DENV serotype with simultaneous circulation of all four DENV serotypes was identified in the study area from December 2015 to March 2017.

**Graphical Abstract:**

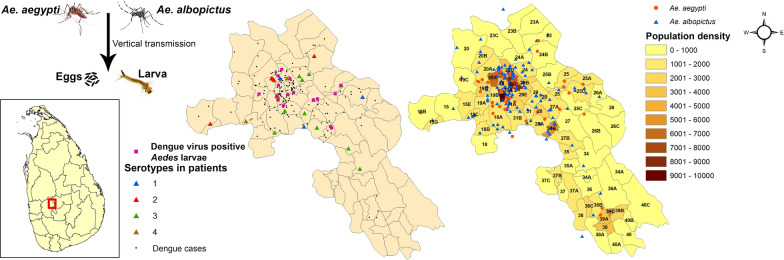

**Supplementary Information:**

The online version contains supplementary material available at 10.1186/s13071-021-05114-5.

## Background

Dengue is a rapidly evolving mosquito-borne viral disease that is endemic in the tropical and subtropical regions of the world [1]. The dengue virus (DENV) is a single-stranded RNA virus belonging to the family *Flaviviridae* and genus *Flavivirus* consisting of four different serotypes: DENV-1, DENV-2, DENV-3 and DENV-4 [2]. The virus is transmitted to humans through the bite of infected *Aedes* mosquitoes, *Aedes aegypti* and *Aedes albopictus*. *Aedes aegypti* is originally from Africa and *Ae. albopictus* is from Asia, but both species have extended their global range to include a number of different countries. In recent times, *Ae. albopictus* expanded its distribution from urban to rural areas with the movement of people from urban to rural areas in tropical countries [3]. Over the past 50 years, the incidence of dengue worldwide has increased by 30-fold, with 390 million dengue cases reported annually according to the World Health Organization (WHO) [4]. Clinical manifestations of dengue range from asymptomatic to life-threatening complications such as dengue haemorrhagic fever (DHF) and dengue shock syndrome (DSS). In 2009, a new classification was proposed for the clinical characterization of dengue, with dengue with and without alarm signs and severe dengue replacing the traditional classification that grouped cases of the disease as dengue fever, DHF and DSS, respectively [4]. DENV was first identified in Japan in 1943, and the earliest known outbreak of DHF occurred in Manila, the Philippines in 1953/1954. The virus has geographically expanded its distribution from south-east Asian countries to Asian countries, including Sri Lanka, India and Pakistan [1, 3, 5].

Sri Lanka has a tropical climate characterized by two rainy (monsoon) seasons. Dengue is a major public health problem in the country which experiences regular outbreaks of the disease [2, 6]. Dengue-like illness has been recorded in Sri Lanka since the beginning of the century, with the first serological confirmation reported in 1962 [7]. Dengue has been considered to be endemic in the country since 1989, with a steady increase in severe dengue and case fatality rates. Initially, dengue cases were seen mainly in and around Colombo, the capital city of Sri Lanka, but the disease has progressively spread to other parts of the country with a resulting increase in its incidence [7–9]. All four serotypes of DENV have been co-circulating in Sri Lanka for more than three decades, with some changes appearing within the serotypes, creating new genotypes [8, 9]. A periodic shift in the circulating DENV serotypes was seen from 1989 to 2008; however, epidemics in the latter part of this period were associated with DENV-3, and the predominant serotype involved in the 2009 dengue epidemic was DENV-1 [9]. From 2012 to 2014, DENV-1 was the predominant serotype contributing to the outbreaks, with a lesser involvement of DENV-4 [10, 11]. In 2017, the predominant circulating serotype was DENV-2, and this serotype contributed to the largest ever dengue outbreak recorded in the country [12].

Entomological surveillance has identified both *Ae. aegypti* and *Ae. albopictus* in different parts of Sri Lanka [6, 13, 14]. Understanding the vectorial capacity of *Ae. albopictus* on DENV transmission would help dengue control measures in suburban and rural areas of the country. The ability of *Ae. albopictus* to survive at diverse tropical and subtropical temperatures ranging from 25 °C to 30 °C and in changing environments may facilitate DENV transmission for an extended period when compared to *Ae. aegypti* [6, 9]. However, it has been argued that *Ae. albopictus* has not played a part in the explosive dengue epidemics and that in comparison to *Ae. aegypti*, it plays only a minor role in DENV transmission [6, 9].

The ability of *Aedes* mosquitoes to transmit the virus to their offspring, known as vertical transmission (VT), may contribute to the persistence of the virus in the environment in the absence of susceptible hosts or during unfavourable environmental conditions [13–16]. Verifying this phenomenon is necessary to understand the dynamics of DENV transmission. Furthermore, changes in the circulating DENV serotypes in a defined geographical area may be dependent on the rate of VT. The aim of the present study was to analyse the percentage of VT in *Aedes* mosquito larvae and in circulating DENV in patients and *Aedes* mosquito larvae across both outbreak and inter-outbreak seasons in an area of Sri Lanka at high risk for dengue.

## Methods

### Study setting

**Fig. 1:**
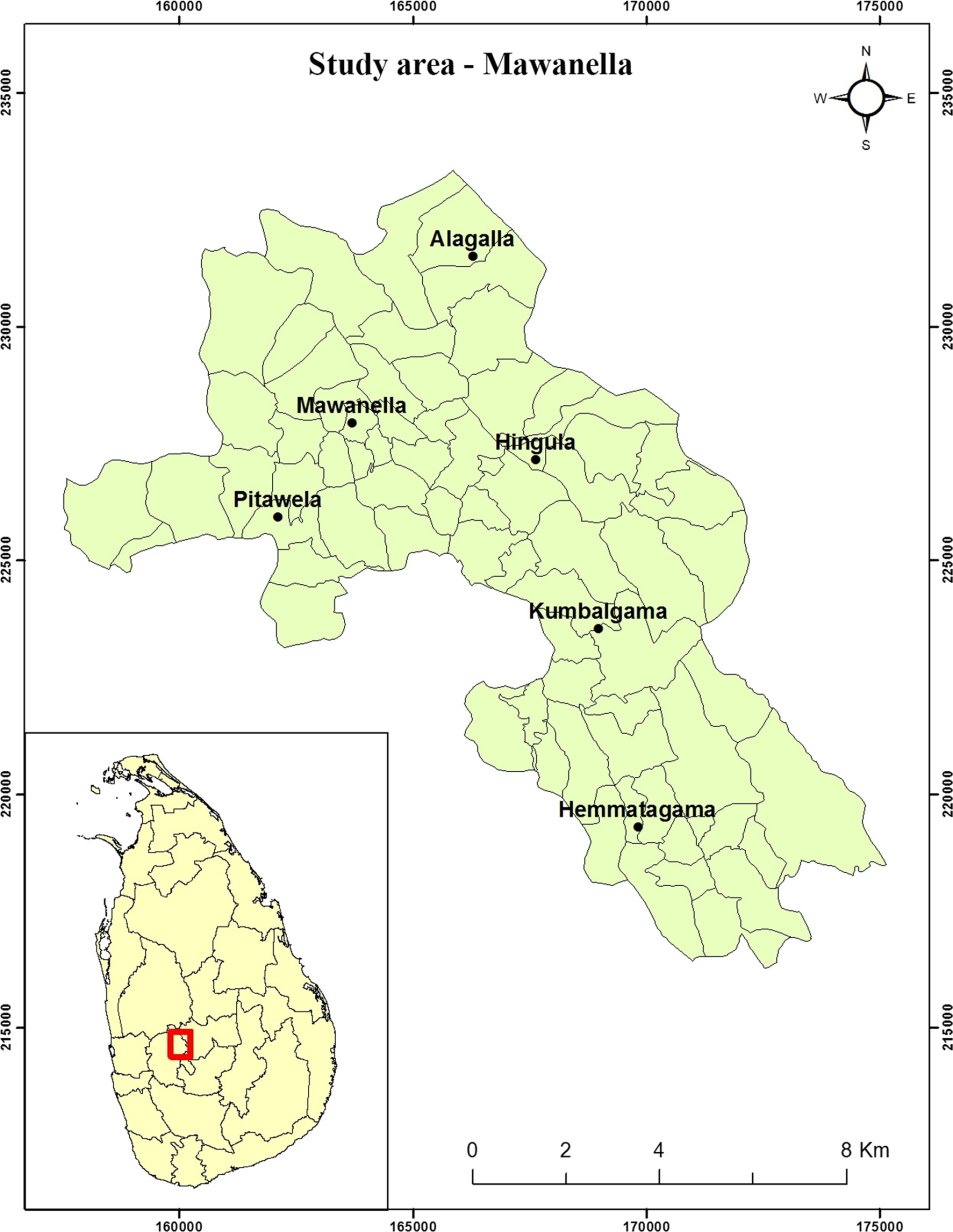
Map of Sri Lanka showing the location of Mawanella, the study area

The study area, Mawanella (Fig. 1), one of the highest dengue risk areas of Sri Lanka, is situated on the south-west side of the tropical mountainous region of central Sri Lanka. The altitude of the area varies between 180 and 260 m a.s.l. The average annual rainfall is 2500–4500 mm, the temperature of the area ranges from 22 °C to 35 °C and the relative humidity of the area ranges from 60% to 90%. The area covers 118 km^2^ of land, with a population size of 1,10,000, giving a population density of 947 people/km^2^. Dengue cases have been reported in the area in the past 15 years, with two peaks from May to July and from October to December, respectively. The study area includes both urban areas with a built-up environment and infrastructure and rural areas with vegetation, rubber estates and wild animals that all provide suitable breeding places for both *Ae. aegypti* and *Ae. albopictus* mosquitoes [3, 14]. The Base Hospital, Mawanella, is the tertiary care hospital of the area, with medical specialists and all essential facilities, and it is the referral centre for patients from small hospitals. All patients with dengue in the study area are treated at the base hospital. Consequently, patients treated in the base hospital, Mawanella, are considered to represent dengue patients in the study area.

### Study design and collection of patients’ data and samples

This is a descriptive cross-sectional study covering patients with clinically suspected dengue who were admitted to the Base Hospital, Mawanella, Sri Lanka from December 2015 to March 2017. A convenient sample size of 200 blood samples was collected from dengue suspected patients for testing in the laboratory to identify the infecting DENV serotype using a reverse transcription PCR (RT-PCR) [17]. Patients were selected from the admission register of the hospital and numbered from the study initiation date. Based on the annual average of dengue cases reported from the study area (*n*  = 300), we estimated that 400 dengue cases would be recruited for the study over a period of 16 months. However, due to financial constraints, only 200 cases were sampled by including every second patient with suspected dengue who was admitted to the hospital from the start of the study.

Data on patients’ residential address, presenting symptoms, clinical diagnosis and non-specific laboratory findings were collected using a standard data collection form. All patients included in the study were aged > 18 years. Of the 200 patients, 153 were male and 47 were female. These patients were suspected of having dengue based on both clinical and non-specific laboratory findings, such as white blood cell and platelet counts on admission, as stated in the WHO guidelines [4].

Blood samples were drawn from suspected dengue patients who were selected based on the most recent case definition for dengue as defined by the WHO [4]. An additional 3–5 ml of blood drawn from each patient by nursing staff  was collected in a plain test tube for research purposes; each patient from whom blood taken for routine investigations on admission provided written consent at the time of blood collection. These samples were centrifuged (1800 *g*, 5 min) to separate the serum, and each serum sample was kept at − 70 °C in the hospital until transported to the Diagnostic and Research Virology Laboratory, Department of Microbiology, Faculty of Medicine, University of Peradeniya where the RT-PCR tests were performed.

### Collection, identification and pooling of *Aedes* mosquito larvae

Entomological surveys were carried out to collect *Aedes* larvae over an area of 400-m radius around patients’ residences; this  size of sampling area was chosen taking the flying range of the *Aedes* mosquitoes into consideration. Houses of patients were visited within 7 days of hospital admission to collect the entomological data, with consent of the occupants. These visits were performed by a team comprising a trained entomological assistant and two to three larvae collectors. All possible collection containers were inspected, including artificial water collection containers, such as discarded utensils, tires, roof gutters and water tanks, and small natural water containers, such as coconut shells and tree trunks.

During the inspections, if ≥ 10 *Aedes* larvae (included first to fourth instar larvae [L1–L4]) were present in a breeding place, a maximum of 10 larvae were collected from each breeding place into a labelled larval collection vial. If there were < 10 larvae in a breeding place, all larvae present were collected and placed into the collection vial [14]. The collection vials were brought to the Entomology Laboratory of the Regional Malaria Office of the Kandy District, Sri Lanka, to identify the  3rd and 4th larval stages using the standard identification keys [18, 19]. L1 and L2 larvae were allowed to develop into L3 and L4 prior to identification. Larvae were then separated into *Ae. aegypti* and *Ae. albopictus* pools under the same identity number as that of the patient. *Aedes* larval pools were marked with the home address of the patient and matched with the blood sample of the patient with the corresponding identification number (that had been assigned in the hospital). *Aedes* larval pools were stored at − 70 °C until the samples were transported to the Laboratory for testing for DENV.

### DENV detection in patients and *Aedes* mosquito larval pools

Viral RNA was extracted from patients’ sera and larvae using the QIAamp Viral RNA Mini Kit (Qiagen, Hilden, Germany) according to the manufacturer’s instructions. When viral RNA was extracted from the larval pool, a 5-h lysis step was performed to allow for the complete digestion of immature larval tissues. The maximum and minimum number of larvae in a larval pool was 70 and 6, respectively. The infection rate in larvae was not calculated.

All RT-PCR tests were performed using a conventional two-step RT-PCR method in a Swift MaxPro Thermal Cycler (Esco Healthcare, Singapore) with primers targeting the capsid region of the virus [17]. DENV-positive samples were then subjected to DENV serotyping using primers targeting the envelope region of the virus [17].

### Statistical analysis

The graphs for the study were constructed using MS Excel 2013 (Microsoft Corp., Redmond, WA, USA). Statistical analysis was done using the SPSS version 16.0 software package (SPSS IBM Corp., Armonk, NY, USA). The difference between the proportions of DENV-positive *Ae. albopictus* and *Ae. aegypti* mosquitoes was analysed using the Fisher’s exact test, and the difference was considered to be significant at *P* value ≤ 0.05. The difference between the proportions of *Aedes* larval (*Ae. albopictus* and *Ae. aegypti*) pools identified during the outbreak and inter-outbreak periods was analysed using the Chi-square test; the difference between proportions of *Aedes* larval pools identified were considered to be significant at *P* ≤ 0.05.

## Results

### DENV infection in patients

**Fig. 2:**
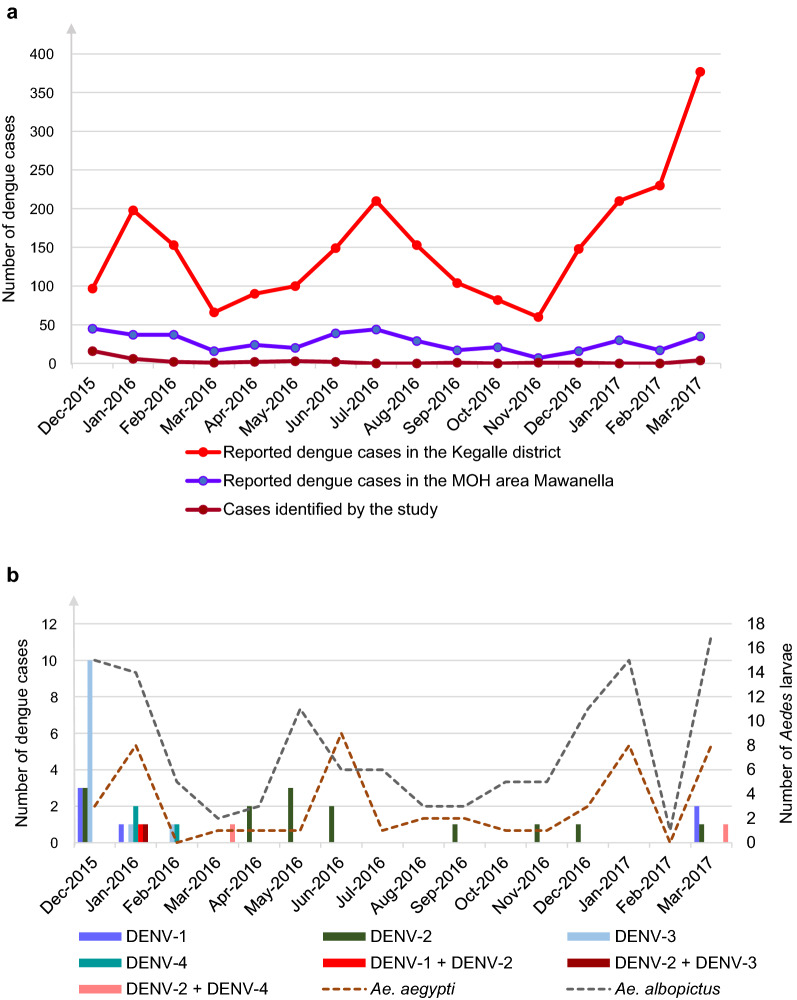
**a** Distribution of dengue cases in the Kegalle District and Mawanella MOH area, Sri Lanka from December 2015 to March 2017. **b **Distribution of DENV serotypes in patients and distribution of *Aedes* mosquito larvae in and around the residences of dengue patients in Mawanella from December 2015 to March 2017. Abbreviations: DENV1, -2, -3, -4, DENV serotypes 1, 2, 3, 4

A total of 200 patients with clinically suspected dengue were studied during the outbreak (December 2015–January 2016, June–August 2016 and December 2016–January 2017) and during the inter-outbreak periods (February–May 2016 and September–November 2016), as shown in the Additional file 1: Table S1. During the study period, the number of dengue suspected cases progressively decreased in comparison to the number of cases reported in the previous years. The incidence of dengue reported in the Kegalle District and Mawanella Medical Officer of Health (MOH) area and study derived dengue incidence in the study area are given in Fig. 2.

All four DENV serotypes were identified in samples from patients with clinically suspected dengue collected from December 2015 to March 2017. At the beginning of the study, from December 2015 to February 2016 (epidemic period), DENV-3 was the most prominent serotype circulating in the study area. DENV-3 was subsequently replaced by DENV-2, and there were four co-infections identified at the beginning (epidemic period) and at the end of the study period (inter-epidemic period) with DENV-2 and other serotypes in patients. DENV-2 was also identified throughout the study period although the number was lower in the early part of the study, before it became the predominant type in the latter part of the study period (March 2017).

### DENV infection in *Aedes* mosquito larvae

*Aedes* mosquito larvae were identified in 171 of 200 entomological surveys conducted from December 2015 to March 2017 in the neighbourhoods of patients; of these, 122 were *Ae. albopictus* larvae and 49 were *Ae. aegypti* larvae. The number of entomological surveys in which *Aedes* larvae were identified was higher during outbreak periods than during the inter-outbreak periods (Table 1; Fig. 2).

**Table 1:** Distribution of *Aedes* mosquito larvae in and around residences of patients with suspected dengue in Mawanella from December 2015 to March 2017

Period	Month and year of sample collection	Total no. of vector pools collected in entomological survey	No. of *Aedes* mosquito pools identified	
			*Ae. aegypti*	*Ae. albopictus*
Epidemic	12/2015	18	3	15
	1/2016	22	8	14
Inter-epidemic	2/2016	5	0	5
	3/2016	3	1	2
	4/2016	4	1	3
	5/2016	12	1	11
	6/2016	15	9	6
Epidemic	7/2016	7	1	6
	8/2016	5	2	3
	9/2016	5	2	3
Inter-epidemic	10/2016	6	1	5
	11/2016	6	1	5
Epidemic	12/2016	14	3	11
	1/2017	23	8	15
Inter-epidemic	2/2017	1	0	1
	3/2017	25	8	17
Total		171	49	122

All 122 pools of *Ae. albopictus* and 49 pools of *Ae. aegypti* were tested to identify the infecting DENV serotypes. Of these, 9.8% (12/122) of *Ae. albopictus* and 8.1% (4/49) of *Ae. aegypti* pools were positive for DENV serotypes (Table 2). The difference between the number of DENV-positive *Ae. albopictus* larval pools and the number of *Ae. aegypti* pools was statistically significant (Fisher’s exact test, *P*  < 0.05).

**Table 2:** Distribution of DENV serotypes in patients with suspected dengue and in *Aedes* mosquito larvae

Patient no.	*Ae. aegypti*	*Ae. albopictus*	DENV serotype identified in mosquito pools	DENV serotype identified in patients
1	Detected	ND	DENV-3	DENV-3
2	ND	Detected	DENV-1	DENV-1
3	ND	Detected	DENV-3	DENV-3
4	ND	Detected	DENV-4	ND
5	ND	Detected	DENV-3	ND
6	ND	Detected	DENV-1	ND
7	Detected	ND	DENV-2	ND
8	Detected	ND	DENV-1	ND
9	Detected	ND	DENV-1	ND
10	ND	Detected	DENV-3	ND
11	ND	Detected	DENV-2	ND
12	ND	Detected	DENV-2	DENV-1
13	ND	Detected	DENV-2	ND
14	ND	Detected	DENV-4	ND
15	ND	Detected	DENV-1	ND
16	ND	Detected	DENV-2	DENV-2

*ND* Not detected

All four DENV serotypes were identified in larvae collected during the entomological surveys conducted from December 2015 to March 2017. Of the 16 DENV-positive *Aedes* larval pools that were identified in the 171 surveys, DENV-1 (31.2%) and DENV-2 (31.2%) were identified in five pools each, DENV-3 (25%) was identified in four pools and DENV-4 (12.5%) was identified in two pools. DENV co-infections were not identified in any of the larval pools tested. All four DENV serotypes were identified across the 12 larval pools that tested positive for *Ae. albopictus* larvae (12/16 *Aedes*-positive pools; 75%); only DENV-1, DENV-2 and DENV-3 were identified in the four larval pools that tested positive for *Ae. aegypti* (4/16 *Aedes*-positive pools; 25%)

The relationship between the infecting DENV serotypes of mosquito larval pools and corresponding patients was also assessed. Of the 16 larval pools positive for DENV, five were positive for the same DENV serotypes infecting the corresponding patient whereas one was different from the serotype infecting the patient.

*Aedes aegypti* mosquito larval pools were found to be more common in inter-outbreak periods (29/49 larval pools) than in the DENV outbreak periods (20/49). The same was true for *Ae. albopictus* mosquito larval pools found between inter-outbreak (65/122 larval pools) and outbreak (57/122) periods. However, the difference between the proportions of *Aedes (Ae. albopictus* and *Ae. aegypti*) larval pools between outbreak and inter-outbreak periods was not statistically significant (Chi-square test, *X*^2^ = 0.28, *df* = 1, *P* = 0.594).

## Discussion

One part of the present study is based on the analysis of 200 patients with clinically suspected dengue who were enrolled in the study during dengue outbreak (December 2015–January 2016 and June–August 2016) and inter-outbreak (February–June 2016, February–March 2017 and September–November 2016) periods. The regular dengue pattern in Sri Lanka, including in the Kegalle District and the MOH area of the study site, has two peaks, with the first peak from December to January and the mid-year peak from June to August (Fig. 2). However, we noted a decrease in the number of suspected dengue cases during the study period compared to the case load reported in the study area in previous years. This decrease may be due to recent fluctuations in the weather patterns contributing to changes in the distribution of cases in a given year [9]. Vector control activities undertaken by the health authorities might also contribute to differences in the reported number of suspected dengue cases in a given year [6].

The study also identified the pattern of circulating DENV in a high-risk area during a period covering outbreak and inter-outbreak seasons. A shift from DENV-3 to DENV-2 was noted in the study area, similar to the changes noted in the circulating DENV serotypes in Colombo, the capital of Sri Lanka, in the same period [12]. DENV-2 may have been introduced from Colombo to the study area as there was a larger dengue outbreak in Colombo during the same period and the city is located only 80 km from the study area. Similar findings to those of the present study were reported from a study conducted in Hanoi, Vietnam, in 2014 [20]; in both studies, a change in the circulating DENV serotypes was observed over a period of 16 months. The observed change in the circulating DENV serotype couldn be due to either the movement of people from the study area to other high-risk dengue areas or from other high-risk dengue areas to the study area for different reasons [6, 9, 21]. It has been shown that DENV can be transported to distances of > 500 km via DENV-infected eggs and larvae in containers or through infected individuals [22]. The introduction of DENV-2 to the study area could have occurred through infected people travelling between the study area and dengue hyper-endemic areas, such as Colombo, resulting in the movement of the virus and mosquitoes. However, there was no clear evidence to identify the place where DENV-2 was first detected during the 2017 outbreak in Sri Lanka, and it may have been either Colombo or the study area. Most importantly, the study showed the change in the circulating DENV serotypes in a 16-month period.

In the second part of the study, DENV was identified in both *Ae. albopictus* and *Ae. aegypti* larvae caught in the residences of patients and in the environment surrounding these residences, similar to findings reported in Brazil, Republic of the Congo, Cameroon, central Africa and Florida [23–27]. The most abundant immature form identified in both the outbreak and inter-outbreak periods in the study was the larvae of *Ae. albopictus*. Larvae of *Ae. aegypti* larvae were not observed in some non-outbreak months; however, the results of the present study suggest a trend towards a greater abundance of *Ae. aegypti* with progression of the epidemic. Entomological surveys conducted in a number of other dengue endemic areas of Sri Lanka and some other dengue endemic countries during different time periods also identified *Ae. albopictus* as the predominant vector [14, 22, 28, 29]. In contrast, in selected locations of Colombo, Sri Lanka, Indonesia, Columbia and Argentina, *Ae. aegypti* was identified as the most predominant vector [13, 30–32].

*Aedes albopictus* adapts better to peri-domestic settings with vegetation, and it also has an opportunistic zoophilic feeding behaviour and occasionally takes blood meals exclusively from humans [3, 6, 9]. Being a semi-urban area with enough vegetation-based breeding places and high population density, the present study area is an ideal place for *Ae. albopictus* to dominate over *Ae. aegypti* and transmit DENV to humans. It is also important to consider the environmental factors other than the vectorial capacity of the *Ae. albopictus* in causing outbreaks. *Aedes albopictus* has been involved in large dengue outbreaks in a number of countries, including China [33] and Japan [34].

There was a similarity between the DENV serotypes in patients and those in immature *Aedes* larvae, with the same DENV serotypes observed in patients and *Aedes* mosquito larvae pools collected from their daily environment on four out of five occasions. However, on one occasion DENV was detected in *Aedes* larvae but not in the patient. One possible explanation for the absence of DENV in a patient with suspected dengue might be a misdiagnosis of an early phase of another febrile illness, such as typhus or leptospirosis, both of which are also prevalent in Sri Lanka. It is difficult to clinically differentiate these latter two infections and dengue at the early phase of infection due to similar clinical features [17, 21]. Specific test methods, such as the non-structural protein 1 (NS1) test, are not commonly used in the laboratory diagnosis of dengue in many parts of Sri Lanka, and it is known that identifying DENV in *Aedes* vectors is a good early warning signal to predict the circulating DENV serotypes in the environment and, consequently, the clinical severity of the impending dengue outbreak in association with the circulating DENV. Additionally, as there is no effective warning system to predict dengue outbreaks, the detection of DENV in *Aedes* mosquitoes can be used as a more sensitive indicator to predict outbreaks. In alignment with the WHO recommendation, the findings of the present study stress the importance of DENV surveillance to predict impending outbreaks of dengue based on the identification of the circulating DENV. This in turn will help predict the disease severity and accelerate control efforts to minimize dengue incidence in affected areas.

We did not identify DENV-infected *Aedes* mosquito larvae pools during the inter-outbreak periods, possibly due to low rate of infection in immature *Aedes* mosquitoes during the inter-outbreak periods [35, 36]. Future studies need to explore the role of DENV maintenance during inter-outbreak periods in large-scale longitudinal studies in at least two dengue high-risk areas in the country.

Based on the entomological survey, we report 9.8% (12/122) positive pools for DENV in *Ae. albopictus* larvae and 8.1% (4/49) positive pools in *Ae. aegypti* larvae, and the difference in DENV positivity between these larval pools was not statistically significant. The level of VT determined by our study appears to be higher than the VT in *Aedes* larvae reported in a recent study by Goncalves et al. [37] where the VT rate was < 3%, a rate too low to be of epidemiological relevance. Torres-Avendaño et al. [38] observed 22% DENV infection rates from VT, and as we did not calculate the infection rates from VT in our study we have no value for comparison. When VT increases in a given area, dengue outbreaks become more difficult to control [39]. In such environments, methods targeting the elimination of adult mosquitoes become ineffective in controlling outbreaks [40], as supported by findings on Zika virus infection showing longevity in the *Aedes* mosquito larvae associated with the Zika virus [41]. Although this has not been shown in DENV-infected larvae, it likely occurs these larvae as well. Larvae are easier, safer and cheaper to sample than adult mosquitoes and thus sampling and identifying the premature stages are more efficient in terms of the early detection of viral presence [42]. Eliminating vertically infected larvae from the endemic areas by monitoring the infection rates in larvae may be used as a novel strategy to control dengue in high-risk areas [43].

As VT facilitates the sustainability and co-circulation of DENV serotypes in high-risk areas, high frequencies of VT could be both a source and a consequence of DENV persistence [42]. Changes noted in the predominant DENV serotypes in the study area might be due to the simultaneous co-circulation of multiple DENV serotypes. Although co-infections of DENV serotypes were not detected in any larval pool, there were differences between the DENV infecting patients and the DENV in mosquito larvae collected from the residential area. Moreover, all four DENV serotypes were found in the study area, a relatively small area. People are at high risk of developing severe dengue in an area with multiple circulating DENV serotypes due to increased chances for frequent infections [44] than in environments where only a single serotype circulates.

## Conclusions

A change in the circulating DENV serotype was noted in a 16-month period in the Mawanella area of Sri Lanka. All four DENV serotypes circulated simultaneously in the study area with the presence of immature forms of *Ae. albopictus* and *Ae. aegypti* in the patients’ surroundings. A VT of 9.8% was noted in *Ae. albopictus* larvae and a VT of 8.1% was noted in *Ae. aegypti* larvae, and the level of VT determined in the current study is much higher than that reported in *Aedes* larvae elsewhere. The most abundant larval form identified in both outbreak and inter-outbreak periods was *Ae. albopictus* larvae, and immature *Ae. albopictus* showed the potential to transmit all four DENV serotypes. The contribution of immature larvae of *Ae. albopictus* to DENV maintenance during an inter-outbreak period warrants further investigation.

## Supplementary Information

**Additional file 1: Table S1.** Distribution of DENV serotypes in patients in Mawanella from December 2015 to March 2017.

## Data Availability

All data generated or analysed during this study are included in this published article and the supplementary information accompanying this article.

## Abbreviations

DENV: Dengue viruses
DHF: Dengue haemorrhagic fever
DSS: Dengue shock syndrome
MOH: Medical Officer of Health
RT-PCR: Reverse transcription PCR
WHO: World Health Organization
VT: Vertical transmission
