# Supplementary material for: Circulating dengue virus serotypes and vertical transmission in Aedes larvae during outbreak and inter-outbreak seasons in a high dengue risk area of Sri Lanka
Source: Parasit Vectors. 2021 Dec 23;14:614. doi: 10.1186/s13071-021-05114-5 (PMC8705164; doi:10.1186/s13071-021-05114-5)
Supplement: Supplementary file 1 — Additional file 1: Table S1. Distribution of DENV serotypes in patients in Mawanella from December 2015 to March 2017. [file 13071_2021_5114_MOESM1_ESM.pdf]

Title: Circulating dengue virus serotypes and vertical transmission in *Aedes* larvae during outbreak and inter-outbreak seasons in a high dengue risk area of Sri Lanka

Running head: Dengue viruses and vertical transmission in *Aedes* larvae

Chandana Wijesinghe[1,2], Jagath Gunatilake[2,3], PHD Kusumawathie[4], PDNN Sirisena[5], SWPL Daulagala[5], Bushran N Iqbal[5], Faseeha Noordeen[5*]

[1]Teaching Hospital, Peradeniya, Peradeniya 20400, Sri Lanka

[2]Postgraduate Institute of Science, University of Peradeniya, Peradeniya 20400, Sri Lanka

[3]Department of Geology, Faculty of Science, University of Peradeniya, Peradeniya 20400, Sri Lanka

[4]Regional office, Anti Malaria Campaign, Kandy 20000, Sri Lanka

[5]Department of Microbiology, Faculty of Medicine, University of Peradeniya, Peradeniya 20400, Sri Lanka

*Corresponding author

faseehan@pdn.ac.lk; faseeha.noordeen12@gmail.com (FN)

Additional file 1: Table S1. Distribution of DENV serotypes in patients in Mawanella from December 2015 to March 2017.

Period	Month & year of sample collection	No. of DENV positive patients / No. of patients tested	Number of isolations of each DENV serotypes in DENV positive patients						
			DENV-1	DENV-2	DENV-3	DENV-4	DENV-1 + DENV-2	DENV-2 + DENV-3	DENV-2 + DENV-4
Epidemic	12/2015	16/18	3	3	10	0	0	0	0
	1/2016	6/17	1	0	1	2	1	1	0
Inter-epidemic	2/2016	2/5	0	0	1	1	0	0	0
	3/2016	1/3	0	0	0	0	0	0	1
	4/2016	2/6	0	2	0	0	0	0	0
	5/2016	3/8	0	3	0	0	0	0	0
	6/2016	2/13	0	2	0	0	0	0	0
	7/2016	0/7	0	0	0	0	0	0	0
Epidemic	8/2016	0/5	0	0	0	0	0	0	0
	9/2016	1/5	0	1	0	0	0	0	0
Inter-epidemic	10/2016	0/6	0	0	0	0	0	0	0
	11/2016	1/6	0	1	0	0	0	0	0
Epidemic	12/2016	1/14	0	1	0	0	0	0	0
	1/2017	0/21	0	0	0	0	0	0	0
Inter-epidemic	2/2017	0/1	0	0	0	0	0	0	0
	3/2017	4/17	2	1	0	0	0	0	1
	Total	39/200	6	14	12	3	1	1	2
